# Performance of colloidal CdS sensitized solar cells with ZnO nanorods/nanoparticles

**DOI:** 10.3762/bjnano.8.23

**Published:** 2017-01-23

**Authors:** Anurag Roy, Partha Pratim Das, Mukta Tathavadekar, Sumita Das, Parukuttyamma Sujatha Devi

**Affiliations:** 1 Sensor and Actuator Division, CSIR - Central Glass and Ceramic Research Institute, Kolkata 700032, India; 2 CSIR - Network Institute of Solar Energy (CSIR-NISE), New Delhi, India,; 3 Physical and Materials Chemistry Division, CSIR-National Chemical Laboratory, Pune 411 008, India

**Keywords:** DSSC, QDSSC, quantum dot, solar cells, ZnO

## Abstract

As an alternative photosensitizer in dye-sensitized solar cells, bovine serum albumin (BSA) (a nonhazardous protein) was used in the synthesis of colloidal CdS nanoparticles (NPs). This system has been employed to replace the commonly used N719 dye molecule. Various nanostructured forms of ZnO, namely, nanorod and nanoparticle-based photoanodes, have been sensitized with colloidal CdS NPs to evaluate their effective performance towards quantum dot sensitized solar cells (QDSSCs). A polysulphide (S*_x_*^2−^)-based electrolyte and Cu*_x_*S counter electrode were used for cell fabrication and testing. An interesting improvement in the performance of the device by imposing nanorods as a scattering layer on a particle layer has been observed. As a consequence, a maximum conversion efficiency of 1.06% with an open-circuit voltage (*V*_OC_) of 0.67 V was achieved for the ZnO nanorod/nanoparticle assembled structure. The introduction of ZnO nanorods over the nanoparticle led to a significant enhancement of the overall efficiency compared to the corresponding bare nanoparticles.

## Introduction

Dye-sensitized solar cells (DSSCs) using inorganic semiconductors are being investigated as a cost-effective and alternative energy source. In DSSCs, a porous electrode made of a wide band gap semiconductor is required for anchoring dye molecules and transporting photo-injected electrons. Commonly used dye molecules are Ru-based N719 and N3. However, recently, chalcogenide semiconductors such as CdS, InP, CdSe, PbS, CdSe, Sb_2_S_3_ have been explored for replacing the sensitizers in DSSC [1–4]. Thus, there has been a renewed interest in the area of DSSC, resulting in the development of the so-called quantum dot sensitized solar cells (QDSSCs) [5–9]. Due to its natural abundance and comparatively lower cost, CdS, one of the important direct band II–VI semiconductors with a band gap of ≈2.4 eV, has been investigated for this purpose. According to the reported results, CdS-sensitized ZnO QDSSCs exhibit a wide range of efficiencies ranging from 0.06 to ≈4% depending on the photoanode, processing technique and sensitizing mechanism [10–16]. For example, in a pioneering study by Liu et al., 1.40% efficiency was reported with an open-circuit voltage (*V*_OC_) of 0.62 V by using hierarchically structured ZnO [11]. A recent study by Poornima et al., on the other hand, reported a slightly higher efficiency of 1.59% with a *V*_OC_ of 0.74 for Cu-doped ZnO [12]. Many others reported comparatively poorer efficiencies of 0.067% and 0.87% along with lower *V*_OC_ of 0.20 and 0.54 V, respectively [10,13]. Interestingly, in all the above cases, the successive ion-layer absorption and reaction (SILAR) process has been used as the sensitization process for QD sensitization. In addition to SILAR, chemical bath deposition (CBD) has also been used as a CdS sensitization technique. Respective efficiencies of 0.87% and 0.72% with *V*_OC_ of 0.44 V and 0.55 V, have been reported by Zhang et al. and Qi et al., for ZnO nanowires which are noteworthy reports [14–15]. For QDSSCs, a polysulphide electrolyte/Cu_2_S electrode delivered the best performance instead of the regular I^−^/I_3_^−^ electrolyte with a costly Pt-based electrode.

Reports on the synthesis of nanoscale CdS by using organic capping agents, polymers, surfactants, or enzymes reveal that they are not very user friendly techniques [17–23]. Therefore, rather taking a conventional path to synthesizing quantum dots (QDs) we followed a bioinspired strategy to synthesize such systems. In this work, bovine serum albumin (BSA) is selected as a biotemplating agent. BSA is a ubiquitous protein mainly present in blood and is a commonly used reagent in biological studies. Here, we have synthesized functionalized colloidal CdS nanoparticles (NPs) by a sono-chemical synthesis using BSA as a biotemplate [24–26]. BSA can construct surface functionalized colloidal CdS QDs with modified optical properties as compared to its bulk form. This can further be employed to render stable dispersion of CdS NPs without nanoparticle aggregation. Various disulfide bonds, thiol residues and other hydroxyl moieties that exist on BSA can serve as effective surface functionalized groups on CdS surfaces [27]. This may further lead to better anchoring with the metal oxide-based photoanode. Among the alternative single oxide materials investigated so far, ZnO delivers effective performance owing to higher band gap (≈3.3 eV) and high electron mobility (≈200–300 cm^2^V^−1^S^−1^) compared to conventional TiO_2_ [28–29]. Also, the flexibility in morphological control and the possibility of low-temperature applications also indicates that ZnO could be a preferential alternative to TiO_2_. However, ZnO suffers from lack of stability in the acidic Ru-based sensitizers in DSSCs, leading to the formation ZnO/dye aggregates [30]. This can be easily avoided for QDSSCs where the used sensitizer, CdS, is neutral in nature. In our earlier work, we have reported the advantages of using 1D ZnO nanorods compared to nanoparticles in DSSCs using N719 as a photosensitizer [31]. Due to the reduced grain boundaries and direct conjunction pathway, 1D nanorods can diffuse electrons faster than nanoparticles and other morphologies. However, nanoparticles lead to higher surface area than 1D nanorods which can sensitize more CdS at a particular time. In this study, we have used synthesized ZnO-based photoanodes exhibiting two different morphologies: ZnO nanoparticles (ZnO-P) and nanorods (ZnO-R) for fabricating QDSSCs. We evaluated their respective performance after sensitization with colloidal CdS. In addition, by using 1D ZnO nanorods as a scattering layer, improved photovoltaic activity of ZnO upon sensitization with colloidal CdS could be achieved [32–33].

## Experimental

### Materials

The raw material used for the synthesis of Cd(NO_3_)_2_, 3H_2_O, Na_2_S·7H_2_O and ethanol were purchased from Merck Limited, Germany. Bovine serum albumin was purchased from Sigma-Aldrich, USA. Zn(NO_3_)_3_ and Zn(CH_3_COO)_2_ were purchased from Merck Limited, Germany. All the chemicals were used without any further purification.

#### Synthesis of CdS nanoparticles

The CdS NPs were synthesized using a BSA-mediated process under sonication. Aqueous solutions of 0.1 M Cd(NO_3_)_2_·4H_2_O and 1.25 × 10^−6^ M (BSA) were sonicated (ultrasonic probe, Rivotek, 30 kHz, 250 W) for 45 min followed by immediate dropwise addition of 0.1 M Na_2_S until the pH of the resultant solution reached ≈4. The sonication was continued for 1.5 h. The yellowish-orange precipitate was centrifuged at 10,000 rpm followed by thoroughly washing with ethanol and distilled water. The precipitate was further dried at 70 °C and calcined at 300 °C for 2 h. The resultant yellowish-orange powder was used for further studies. ZnO nanoparticles and nanorods were synthesized by a solution-growth process, the details of which are reported elsewhere [31–32].

#### Fabrication of CdS-NP-sensitized ZnO-based films

ZnO nanoparticle (ZnO-P) and nanorod (ZnO-R) films were fabricated by the doctor blade method on FTO glass (7 Ω/cm^2^) and annealed at 450 °C for 30 min. In addition, a ZnO-R layer was deposited over the ZnO-P layer to fabricate a ZnO nanoparticle/rod (ZnO-P+R) assembled film. For making CdS coatings on the ZnO photoanode films by CBD process, the fabricated films were dipped in 0.5 M of synthesized colloidal CdS dispersion in ethanol (pH 6.4) for 18 h under constant stirring at room temperature. Subsequently, the fabricated films were thoroughly washed with ethanol and dried at 60 °C. The fabricated photonaode films were further employed for in-situ CdS sensitization by the SILAR process. Here, the films were successively dipped in 0.1 M Cd (NO_3_)_2_ and 0.1 M Na_2_S solution in ethanol for 30 s. The cycle was repeated five times and finally the CdS deposited films were thoroughly washed with ethanol. An effective area of 0.25 cm^2^ (0.5 × 0.5 cm) was selected for measurement with proper sealing. Two different types of electrolytes were used for the cell fabrication, such as polysulphide and iodine/iodide. The electrolyte solution was prepared with 0.5 M Na_2_S, 1 M S and 0.02 M KCl in ethanol: water mixture (7:3) solution. In addition, to fabricate the counter electrode, a 0.1 M Cu(NO_3_)_2_ solution in ethanol was drop-casted followed by 0.05 M Na_2_S solution in ethanol on the FTO glass (7 Ω/cm^2^) and fired at 420 °C for 20 min. [34–35]. In some cases, a platinum counter electrode was also used.

#### Characterization of CdS NPs

The structural properties of dried CdS powder were characterized using X-ray diffraction (XRD) analysis on an X’pert pro MPD X-ray diffractometer by PANanalytical with Cu Kα radiation (λ = 1.5406 Å). Fourier transform-infrared (FT-IR) spectra were measured between 4000 and 400 cm^−1^ on a Perkin Elmer, Spectrum Two FT-IR spectrometer with a resolution of 4 cm^−1^ on the dried powder using potassium bromide (FTIR grade ≥99% trace metal basis, Sigma-Aldrich). The as-received KBr was oven-dried overnight at ≈100 °C and then stored in a desiccator prior to use. Furthermore, the Raman spectrum was collected using a 514.5 nm Ar^+^ green laser excitation source with 50 mW power on a STR500, Cornes Technologies system. The ethanol dispersion of the synthesized CdS was used to measure the absorption spectrum on a UV–vis–NIR spectrophotometer (Shimadzu, UV-3600). The emission spectra of the same dispersion was recorded on a steady-state spectrofluorometer (QM-40, Photon Technology International) using a Xe lamp (150 W) as an excitation source with a bandpass of 5 nm at room temperature. The fluorescence quantum yield of the colloidal CdS solution was measured on the steady state fluorometer using an additional integrating sphere [32]. Zeta potential measurements were carried out on a Horiba Nanoparticle Analyzer-SZ100. The morphology of the synthesized powder was monitored on a transmission electron microscope (TEM) with a Tecnai G2 30ST (FEI) high-resolution TEM operating at 300 kV.

#### Characterization and performance evaluation of CdS-sensitized ZnO-R and ZnO-P films

The fabricated films were characterized by structural evaluation using X-ray diffraction analysis on an X’pert pro MPD XRD from PANanalytical with Cu Kα radiation. The microstructural study and cross-sectional thickness of the fabricated films were monitored on a scanning electron microscope (SEM) (LEO 430i, Carl Zeiss). Diffuse reflectance spectra were measured on a UV–vis–NIR spectrometer (Shimadzu, UV-3600) on the considered films. Contact angle (CA) measurements were performed on a KRUSS Drop Shape Analyser, Germany (DSA-4). The photovoltaic *J*–*V* characteristics were measured using a solar simulator (Newport) at 100 mW/cm^2^ (1 sun AM 1.5). A standard silicon solar cell (serial number 189/PVM351) from Newport, U.S. was used as a reference cell. The active area of the fabricated cells used for photovoltaic measurement was 0.25 cm^2^. Further, the *I*–*V* characteristics under normal light on CdS-sensitized ZnO films were measured using an Agilent two-channel precision source and measurement unit (model no. B2902A).

## Results and Discussion

### Structural and microstructural studies of CdS NPs

**Figure 1:**
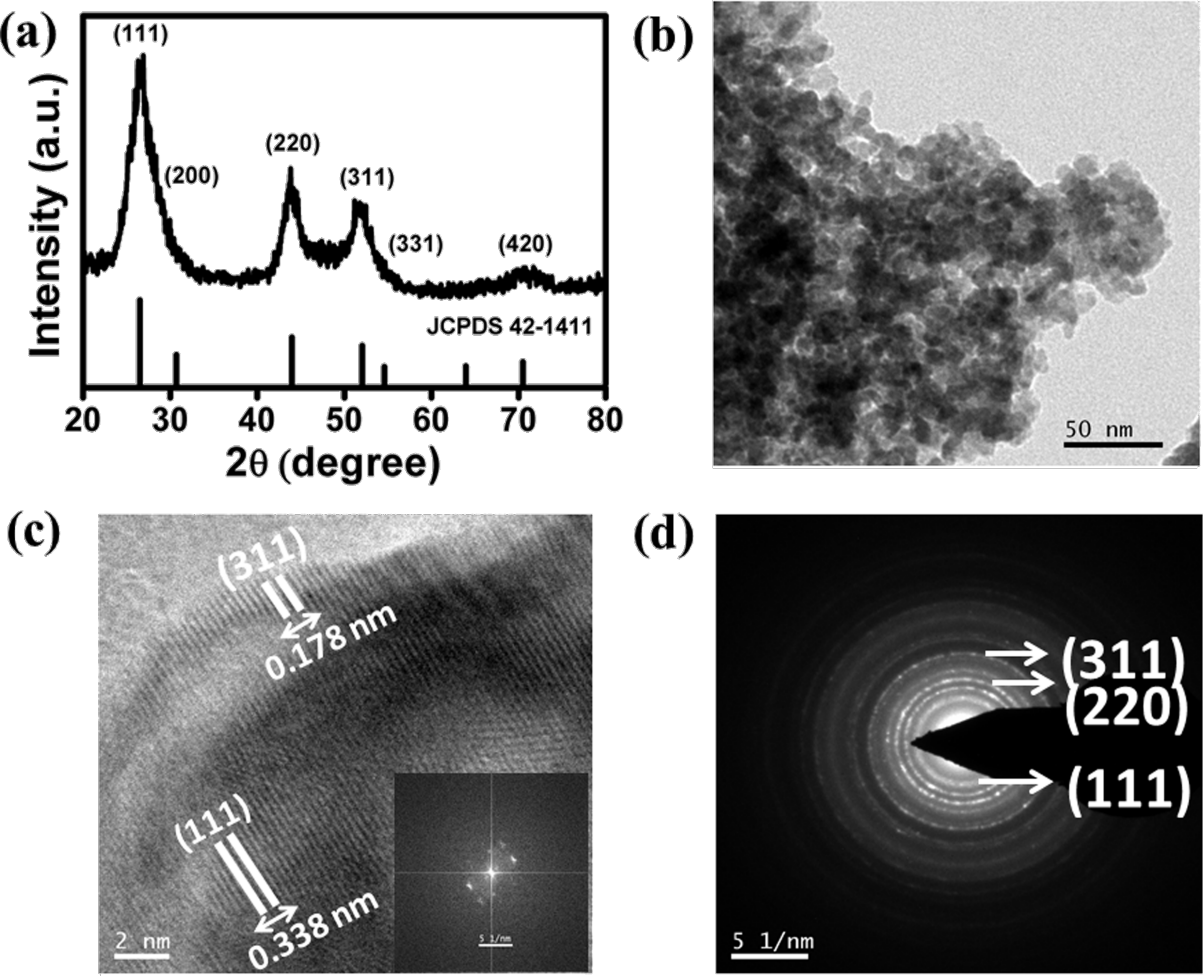
(a) Powder X-ray diffraction pattern of the synthesized cubic CdS powder along with standard JCPDS card no. 42-1411. (b) Bright field transmission electron microscope image of the NPs. (c) HRTEM image highlighting the inter-planar distance of the cubic crystal planes (Inset: corresponding FFT pattern). (d) SAED pattern from image in (b).

The XRD pattern of the synthesized CdS NPs is shown in Figure 1a. All the peaks were well-indexed and matched with the cubic phase of CdS, corresponding to the JCPDS card number 42-1411. Furthermore, the lattice constant was calculated to be 5.585 Å which again matches with the reported value. The broadness as well as intensity of the peaks of the XRD reflections indicates the smaller size of the formed nanoparticles. The mean size of the crystallites was calculated to be ≈5.2 nm using the Scherrer equation. The bright field image of the synthesized powder indicated the finer and porous nature of the synthesized CdS nanoparticles (Figure 1b). The high resolution TEM (HRTEM) image shows the (111) and (311) crystalline planes with *d* values of 0.338 nm and 0.178 nm, respectively, of cubic CdS as shown in Figure 1c. The corresponding FFT pattern is shown in the inset of the figure. The selected area diffraction (SAED) pattern also indicated the major crystalline planes (311), (220) and (111) of cubic CdS as shown in Figure 1d. A quantitative elemental energy dispersive X-ray (EDAX) analysis was performed and the results are given in Supporting Information File 1, Figure S1, to further confirm the formation of CdS.

### Spectroscopic analysis of CdS NPs

**Figure 2:**
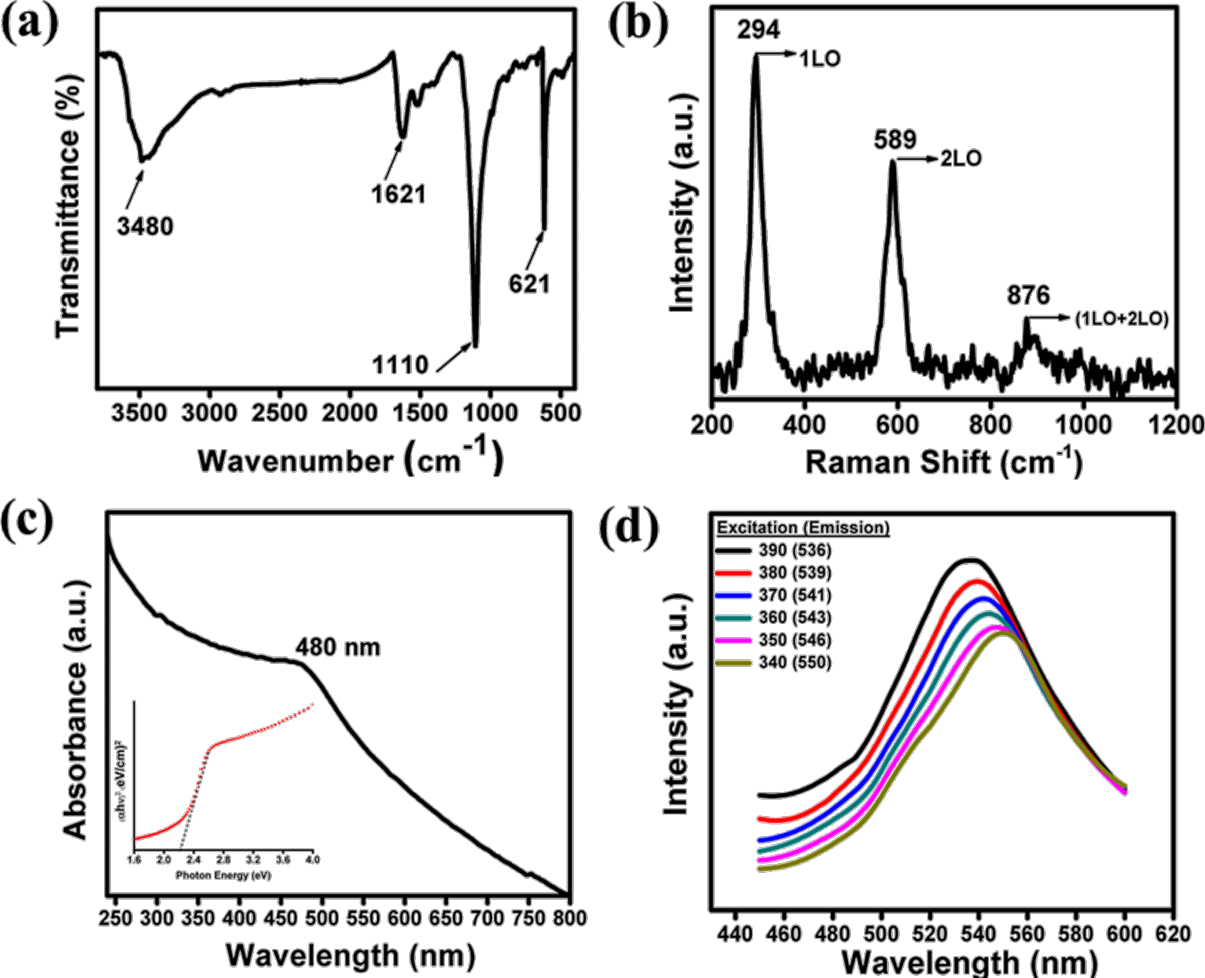
(a) and (b) show the FT-IR and Raman spectrum, respectively, of the synthesized CdS NPs. (c) UV–vis absorption spectrum (Inset: corresponding Tauc Plot for optical band gap measurement) and (d) excitation wavelength dependent emission spectra of the CdS NP dispersion at room temperature.

The FTIR spectrum of the synthesized CdS NPs is shown in Figure 2a. The band at 621 cm^−1^ corresponds to the Cd–S stretching mode. Moreover, the bands assigned at 1110 and 1621 cm^−1^ are attributed to the C–S, S–H and C–O stretching modes, corresponding to the surface functionalized CdS NPs, whereas the band at 3480 cm^−1^ is responsible for labile –OH stretching due to surface adsorbed water [36]. The surface-functionalized CdS acts as an anchoring moiety, which promotes CdS towards effective sensitization with ZnO. The Raman spectrum was performed as shown in Figure 2b. The peaks located at 294 and 589 cm^−1^ correspond to the fundamental band (1LO) and corresponding overtone (2LO) of longitudinal optical (LO) phonon modes of CdS, respectively. The peak 876 cm^−1^ is assigned to a combination of (1LO + 2LO) [37–38].

The UV–vis absorption spectrum of CdS NPs exhibited an absorption edge (λ_e_) at ≈480 nm (Figure 2c) which further depicted an average nanoparticle size of 4.71 nm as derived from Henglein’s empirical equation [39]. The corresponding optical band gap was calculated to be 2.33 eV from the Tauc plot, considering allowed direct transition for synthesized CdS NPs (inset of Figure 2c). The emission spectrum of CdS NPs was monitored at room temperature by varying the excitation wavelength from 340 to 390 nm, as shown in Figure 2d. A green emission at 536 nm was observed against all the excitation wavelengths. The intensity of the emission band was maximum at the excitation wavelength of 390 nm. The solution of synthesized CdS in ethanol resulted in a quantum yield (QY) of 3.116% at room temperature. This is an indication of the ratio of the number of fluorescence quanta to the number of absorbed quanta which is directly proportional to the fluorescence lifetime. Zeta potential (ζ) measurements were also carried out to verify the stability of the CdS NPs in ethanol. A zeta potential value of −38.8 mV (as shown in Supporting Information File 1, Figure S2) illustrates the excellent stability of the dispersion as required for the sensitization resulting in a colloidal solution [40].

### Structural and optical study of CdS films

**Figure 3:**
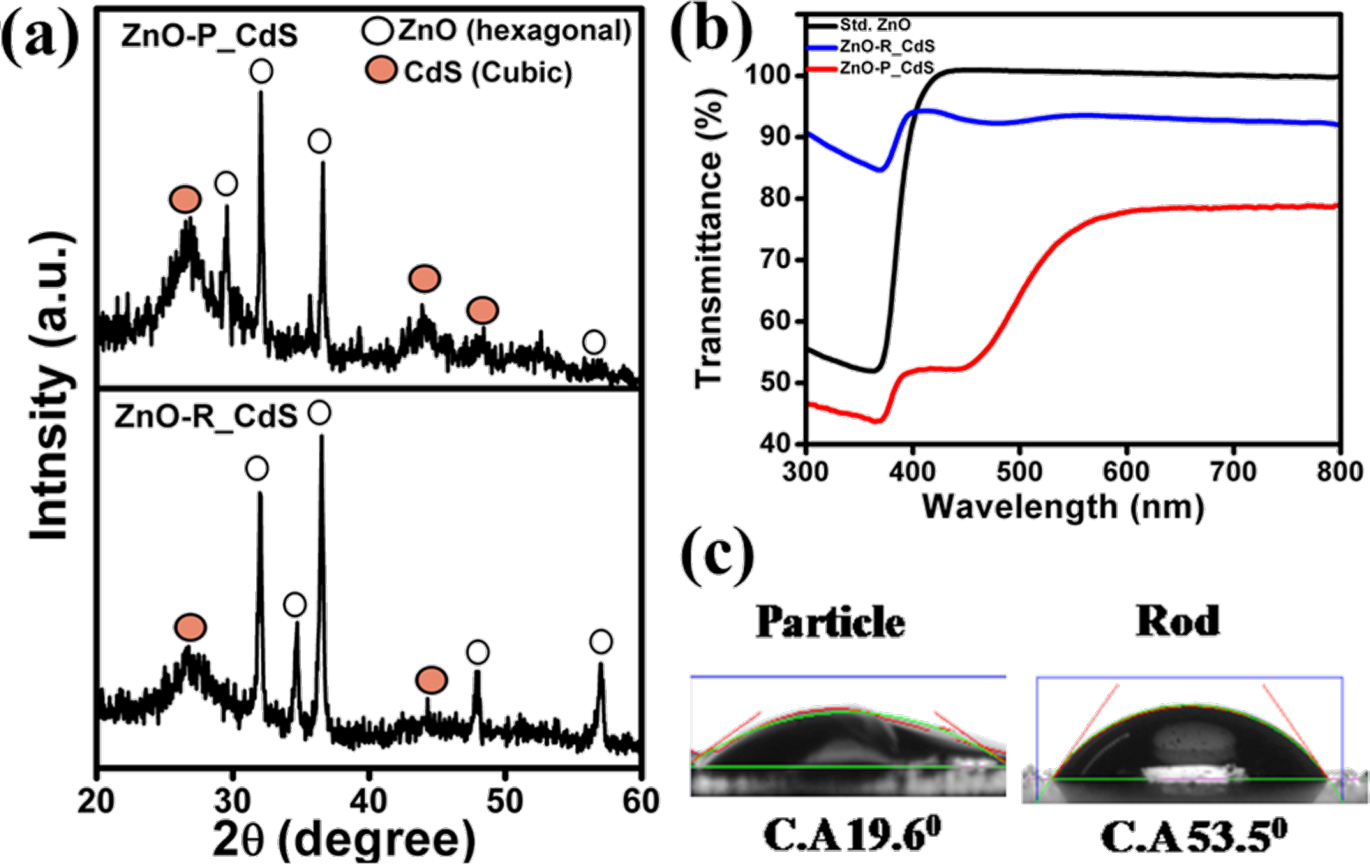
(a) Respective X-ray diffraction patterns of the CdS-sensitized ZnO-P and ZnO-R films. (b) Diffuse reflectance spectrum of the same films compared to bare ZnO and (c) a digital image showing the difference in contact angle for ethanolic solution of N719 dye for ZnO-R and ZnO-P films.

In order to characterize the CdS NPs sensitized on different ZnO films, the X-ray diffraction and diffuse reflection studies of the films were performed. The appearance of the cubic phase of CdS along with expected hexagonal ZnO phase confirms effective sensitization of CdS over ZnO (Figure 3a). This further demonstrates the stability of formed CdS even after the sensitization on the photoanode. It is also interesting to notice the increased intensity of X-ray reflections of CdS after sensitization over ZnO-P compared to ZnO-R. The relatively lower reflectance spectrum of the CdS-sensitized ZnO-P illustrates a better CdS sensitization phenomenon and corroborates the XRD analysis, as shown in Figure 3b. Further investigation of contact angle (CA) measurements by using CdS dispersions on ZnO-based films justifies the sensitization capability. The reduced CA value of 19.6° for the film fabricated with ZnO-P further ascertains its better CdS affinity whereas a moderate CA value of 53.5° was observed for ZnO-R, as shown in Figure 3c.

**Figure 4:**
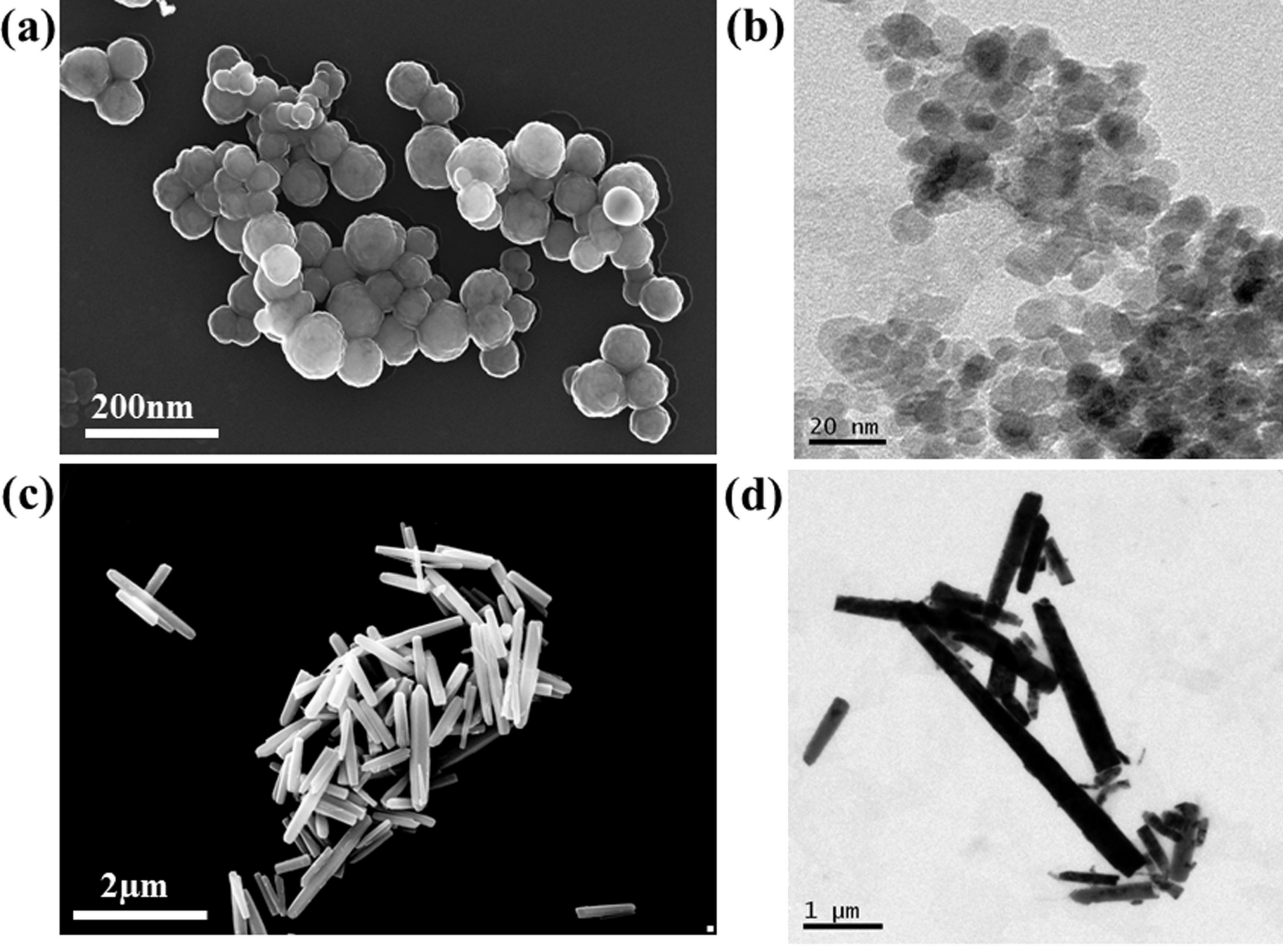
(a,b) Microstructural FESEM and TEM bright field images of synthesized ZnO-P and (c,d) ZnO-R.

The higher surface area of ZnO-P facilitates better CdS sensitization compared to the rod-shaped ZnO, having low surface area and rough surface characteristics. In order to establish the deposition of CdS over ZnO-based films, the FESEM images of the sensitized films fabricated on ZnO-P and ZnO-R were monitored as depicted in Figure 4. The microstructural FESEM and TEM bright field images of the synthesized ZnO-R and ZnO-P are shown in Figure 4. The average nanoparticle size of the ZnO-P was calculated to be ≈12 nm from TEM. It is evident from the SEM images that the formation of ZnO nanorods in the as-prepared stage have an average length of ≈4 μm and a range of diameters between 50–200 nm.

**Figure 5:**
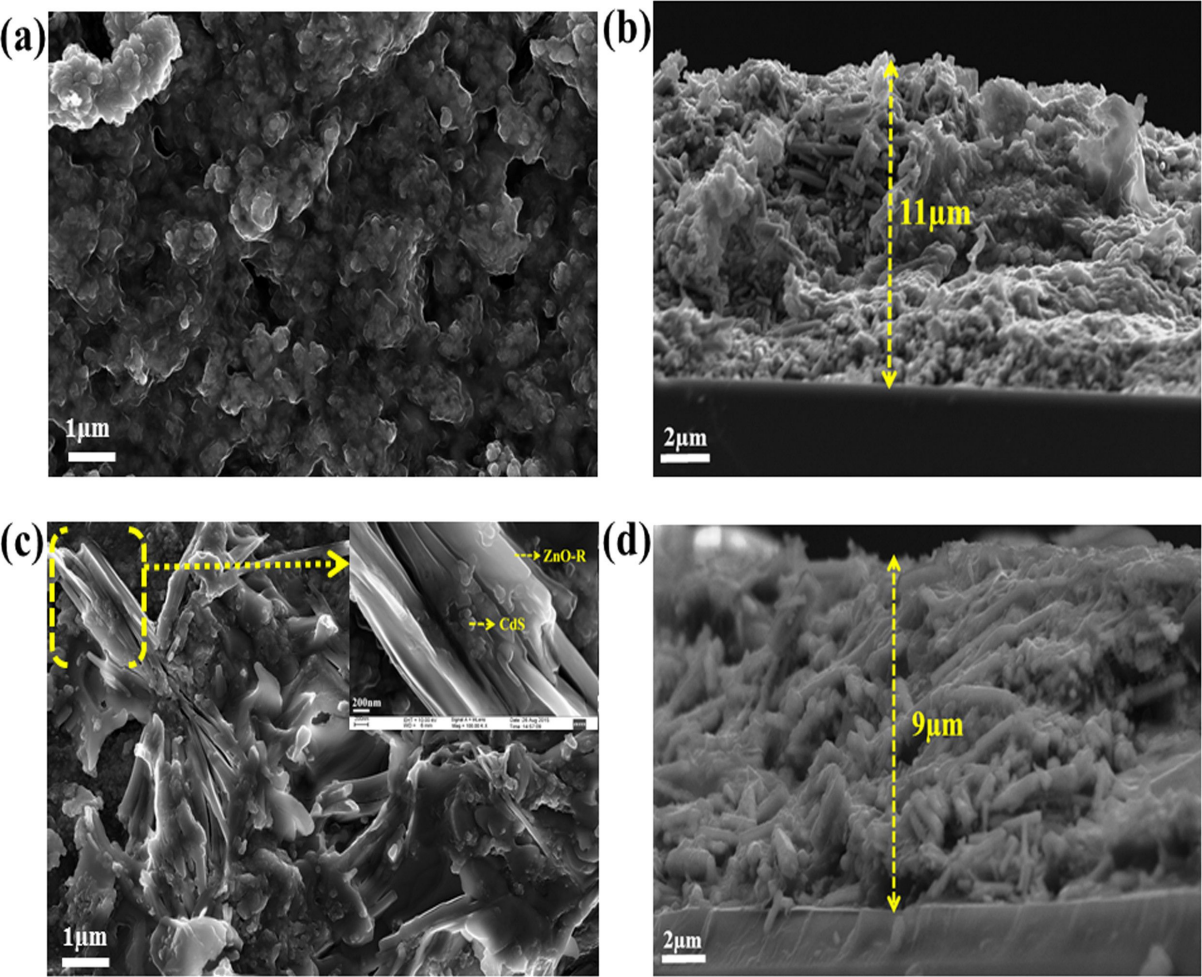
FESEM images of the surface and the corresponding cross-sectional view of CdS NP-sensitized ZnO-P-based film (a) and (b) and ZnO-R based film (c) and (d).

The microstructural images in Figure 5a,c manifest the successful deposition of CdS NPs over the ZnO films. The cross-sectional FESEM images of ZnO-P and ZnO-R films revealed a thickness of ≈11 µm and ≈9µm, respectively, as shown in Figure 5b,d. The aggregation of ZnO-P helped in increasing the film thickness to some extent compared to ZnO-R having irregular orientation. It is worth noting the homogeneous growth of both nanoparticle and nanorod-based nanostructures of ZnO on the FTO surface.

**Figure 6:**
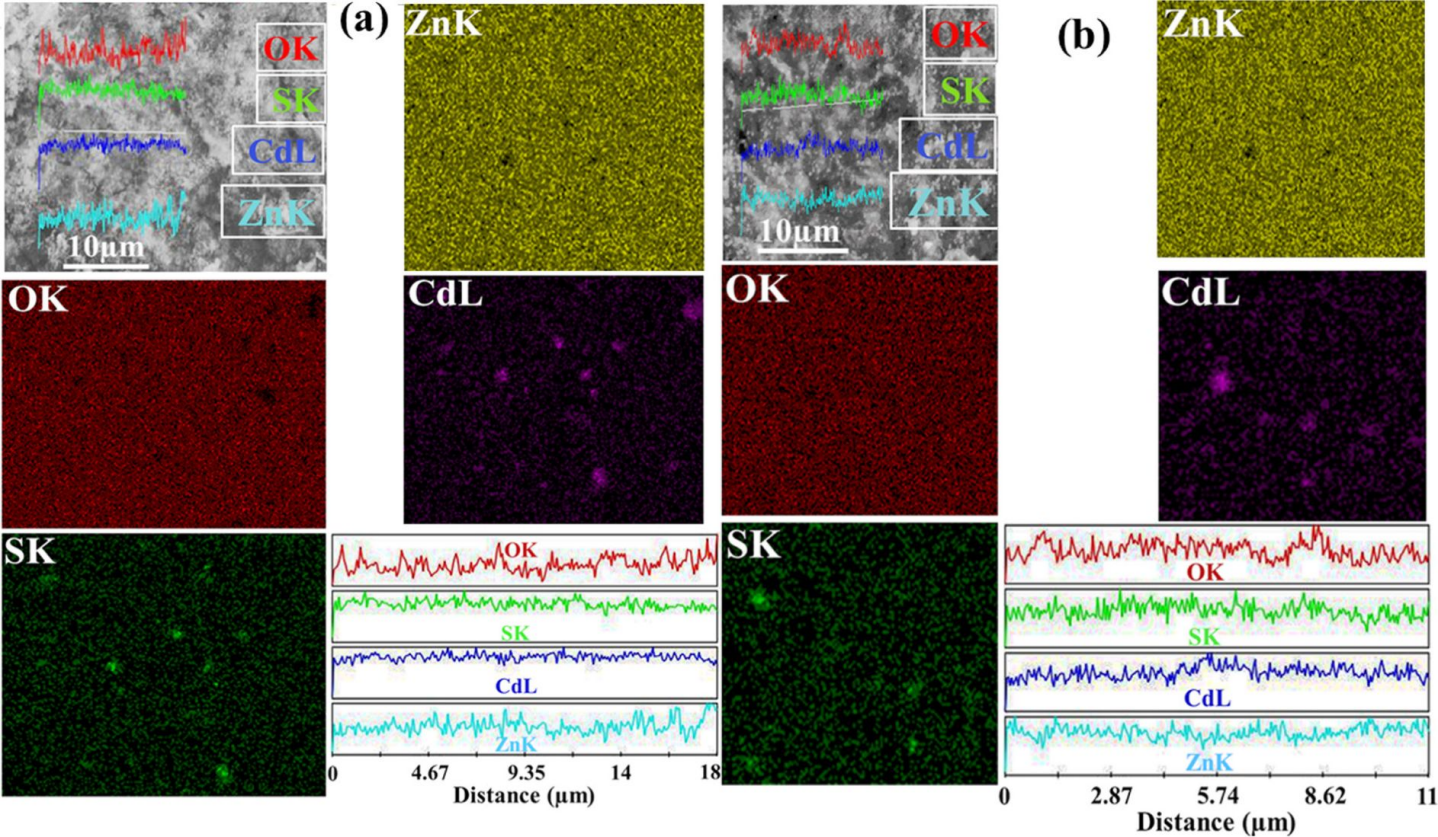
(a) and (b) FESEM elemental and line scale mapping of CdS-sensitized ZnO-P and ZnO-R films, respectively.

The FESEM elemental mapping with distinct color contrast along with line scale mapping were recorded on the sensitized films as shown in Figure 6a,b for the CdS-ZnO-P and CdS-ZnO-R samples, respectively. The homogeneous distribution of Zn and O followed by the coating of CdS is very clear from the elemental mapping diagram. The presence of more CdS is clear on the ZnO-P surface compared to the ZnO-R surface. This confirms the homogeneous distribution of CdS throughout the ZnO surface as illustrated in Figure 6a,b.

### Performance of the fabricated solar cells

**Figure 7:**
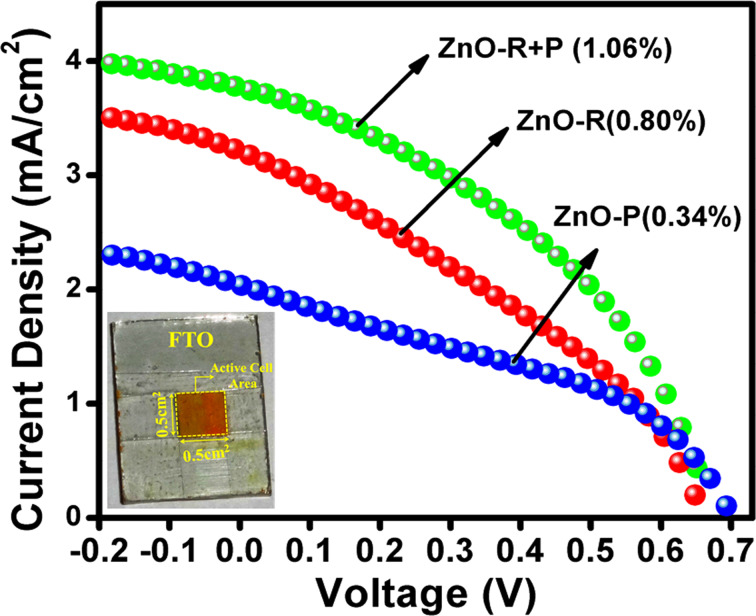
Current density (mA/cm^2^)–voltage (V) curve of CdS-sensitized ZnO-P, ZnO-R and ZnO-R+P based cells. (Inset: digital image of the colloidal CdS sensitized ZnO-R+P film).

The films fabricated with ZnO-P and ZnO-R as the photoanode followed by sensitization with CdS NPs was further characterized by *J*–*V* measurements as shown in Figure 7. ZnO-P based cells exhibited an overall conversion efficiency of 0.34%. In spite of higher CdS sensitization, the undesired agglomeration forms more interparticle gaps. This leads to the higher possibility of electron recombination and entrapment resulting in poor photovoltaic performance for particles. On the contrary, the multioriented 1D ZnO nanorods promote better light harvesting and faster electron injection, resulting in modest improvement in short circuit current (*J*_SC_) as well as fill factor (FF) with an efficiency of 0.80%. On the other hand, for QDSSC systems, the electrolyte and counter electrode combination is another important factor which affects the efficiency of the device. We have tested polysulphide as an electrolyte with a Cu_2_S electrode exclusively for the above measurements. In order to monitor the effect of the popular I^−^/I_3_^−^ electrolyte on the cell efficiency, for the CdS-sensitized ZnO-P and ZnO-R samples, the measurements were also carried out using I^−^/I_3_^−^ electrolyte with an expensive Pt electrode as illustrated in Supporting Information File 1, Table S1. The efficiency is drastically varied upon replacement of the above system with the popular electrolyte. The redox potential of S^2−^/S*_x_*^2−^ and I^−^/I_3_^−^ electrolyte is −0.50 V and −0.35 V vs NHE, respectively. In order to obtain a regenerative redox couple, a second element is needed to couple with S^2−^ from the Na_2_S. In most studies, sulfur is added to the sulfide salt to form a polysulfide (S^2−^/S*_x_*^2−^) redox couple. From the above results it is evident that polysulphide electrolyte is more suitable for achieving better efficiency whereas the I^−^/I_3_^−^ system is better suited for obtaining higher fill factors. The low value of fill factor obtained using the polysulphide electrolyte may be ascribed to the lower hole recovery rate of the polysulfide electrolyte, which leads to a higher probability for charge recombination and loss. In our case, the synthesized ZnO-P exhibits a weak, wide, visible emission from 500–700 nm upon excitation at 345 nm at room temperature as shown in Supporting Information File 1, Figure S4. The visible emission of ZnO basically arises from various surface defects, which can act as photogenerated electron trap states causing a loss of excited electrons [31]. Interestingly, the synthesized ZnO-R does not exhibit any significant visible emission with respect to ZnO-P at the same excitation wavelength. Therefore, we have introduced ZnO-R as a scattering layer to overcome this issue. Thus, we have further fabricated cells employing nanoparticles as a blocking layer for better CdS sensitizing and nanorods as a scattering layer for pronounced light harvesting [31–32,41–42]. A significant enhancement in the photovoltaic performance has been noticed for the ZnO-P+R based cell exhibiting an efficiency of 1.06% (Figure 7), which is remarkably improved compared to ZnO-P. This gives ≈32% enhancement for overall efficiency compared to bare ZnO-R-based cells. In addition, a significant enhancement was noticed for the same film compared to bare ZnO-P based film. This confirms the effective influence of nanorods as a scattering layer and nanoparticles in enhancing the photovoltaic performance of the colloidal CdS sensitized cells. The photovoltaic parameters have been collected in Table 1.

**Table 1:** Photovoltaic parameters of colloidal CdS-sensitized ZnO solar cells.

Cell type	*V*_oc_ ± 0.02 (V)	*J*_sc_ ± 0.01 (mA/cm^2^)	FF ± 0.05 (%)	Efficiency ± 0.03 (%)	CdS form

ZnO-P	0.68	2.00	24.88	0.34	Colloidal CdS
ZnO-R	0.66	3.48	34.13	0.80	Colloidal CdS
ZnO-R+P	0.67	3.75	41.43	1.06	Colloidal CdS
ZnO-P	0.66	0.26	32.81	0.03	SILAR CdS
ZnO-R	0.73	1.03	30.42	0.23	SILAR CdS
ZnO-R+P	0.70	2.54	40.19	0.75	SILAR CdS

A similar set of experiments were carried out following the traditional SILAR process for in situ formation of CdS over ZnO-based films. The thickness of the films has been optimized to be same as for the CBD technique. A comparative study of the photovoltaic parameters for both the sensitization processes is collected in Table 1. Interestingly, a general trend of improvement in photovoltaic performance has been noticed for CBD compared to SILAR process. A probable electronic transition mechanism within the whole fabricated cell is proposed as follows:

In sensitizer:

[1]
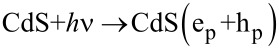


[2]
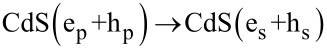


[3]
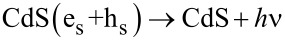


At photoanode:

[4]
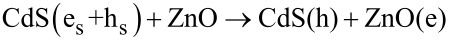


where the subscripts s and p refer to the electronic states of the electrons (e) and holes (h), respectively.

At electrolyte:

[5]
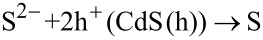


[6]
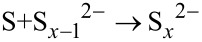


At counter electrode:

[7]
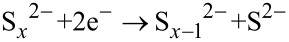


In Table 2, the reported results on CdS-mediated cell performance are compared. Interestingly, a general trend of improvement in photovoltaic performance has been noticed for the ZnO nanorods and wires compared to other nanoforms. In comparison to the reported results on the QDSSCs based on ZnO photoanodes (Table 2), the results obtained in this work are quite competitive and interesting. In addition, the *V*_OC_ and FF of the measured cells are also higher compared to the literature results. This confirms the effectiveness of utilizing the BSA-mediated CdS in improving the QDSSC performance with ZnO as photoanodes. The *I–V* characteristics of the colloidal CdS-sensitized ZnO-P, ZnO-R and ZnO-R+P films measured within the bias voltage −10 V to +10 V in visible light is shown in Supporting Information File 1, Figure S3, exhibiting ohmic response. *I–V* characteristics also sustain higher current generation for 1D ZnO-R than ZnO-P. Further, a significant enhancement in current was observed upon deposition of the nanorods over nanoparticles as ZnO-R+P shows better current conducting behavior for this particular system. As we have confirmed earlier, ZnO nanorods provided more channels for the fast transportation of the photoexcited electrons into the photoanodes, resulting in smaller probability of recombining leading to higher *V*_OC_ and FF.

**Table 2:** Reported performance of CdS-sensitized ZnO-based solar cells.

Photoanode	CdS sensitizing process	Fill factor (%)	Efficiency (%)	Reference

ZnO NPs	SILAR	29.70	0.067	[10]
ZnO nanowire	SILAR	31.49	3.53	[43]
Porous ZnO nanosheets	SILAR	28.00	1.16	[44]
ZnO NPs	SILAR	30.00	0.85	[13]
ZnO nanowire	CBD	29.72	0.34	[14]
ZnO nanorod	Electrodeposition	34.40	1.07	[45]
ZnO nanorod array	CBD	27.00	0.29	[46]
ZnO	SPD	33.00	1.54	[47]
ZnO nanorod	Post-deposition	34.13	0.80	current work
ZnO nanorod/nanoparticle	Post-deposition	41.43	1.06	current work

**Figure 8:**
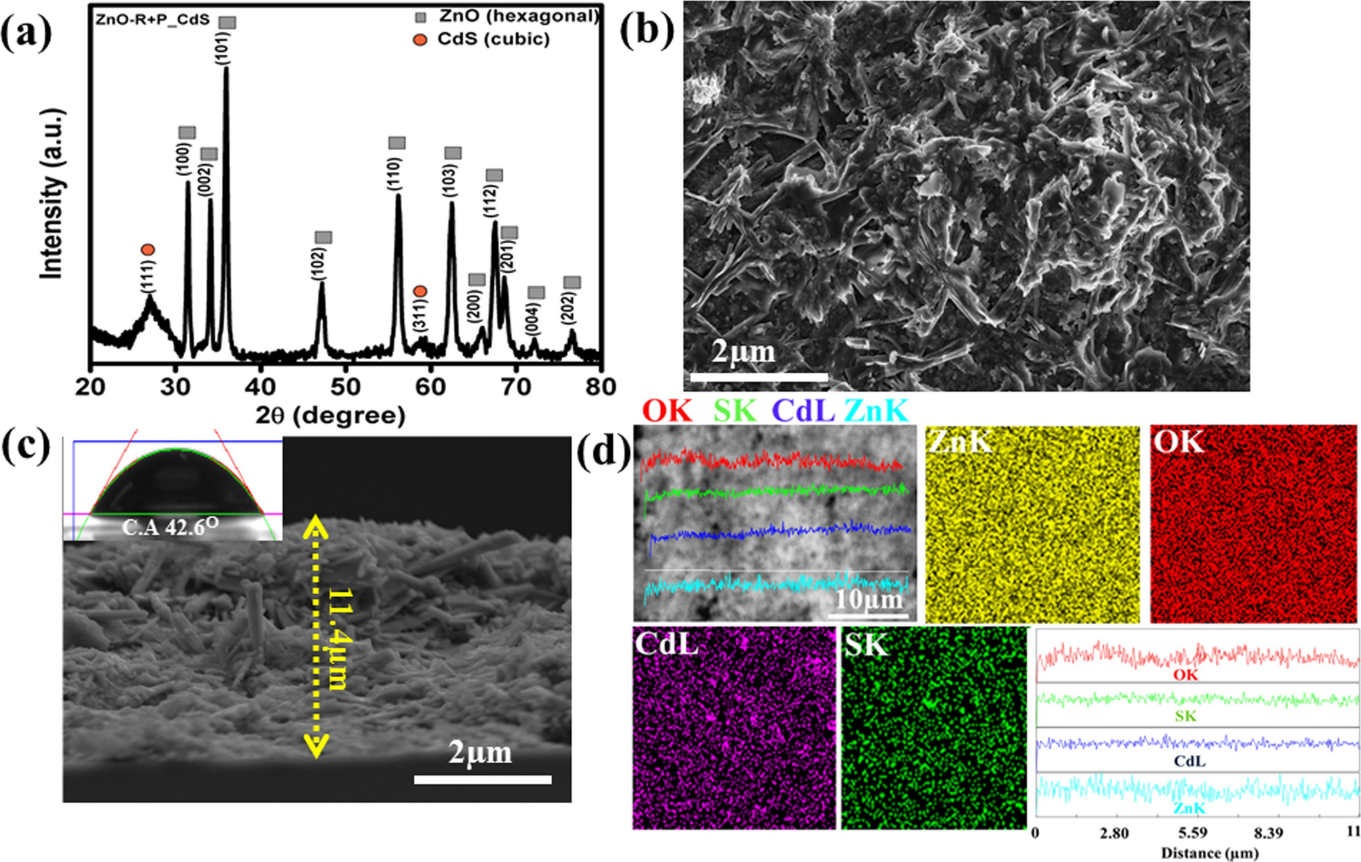
(a) X-ray diffraction pattern of the colloidal CdS sensitized ZnO-R+P based film, (b) corresponding FESEM microstructural image, (c) cross-sectional view of the same film (inset: digital image of CdS contact angle measurement), (d) elemental and line mapping images of the same film.

The X-ray diffraction pattern of the highest efficiency system, ZnO-R+P based CdS sensitized film, is shown in Figure 8a. It also indicates successful CdS sensitization over the film keeping the as-synthesized crystalline phase intact, and it is comparably higher than the ZnO-R film alone. The corresponding FESEM microstructural surface and cross-sectional images are given in Figure 8b,c. From Figure 8b, the surface of the CdS sensitized ZnO-R+P film shows agglomerated nanorods with particles and there is a layer of CdS over them, indicating effective CdS sensitization. Furthermore, the thickness of the final cell was found to be 11.4 µm from Figure 8c, where it is clearly shown that nanorods are randomly distributed over the particle layers in the ZnO-R+P film. Again, the CA value was found to be 42.6° (inset: Figure 8c) which strongly supports a rod-oriented film surface. The elemental mapping along with line scale mapping further confirms the homogeneous distribution of CdS throughout the ZnO-R+P surface, as illustrated in Figure 8d.

**Figure 9:**
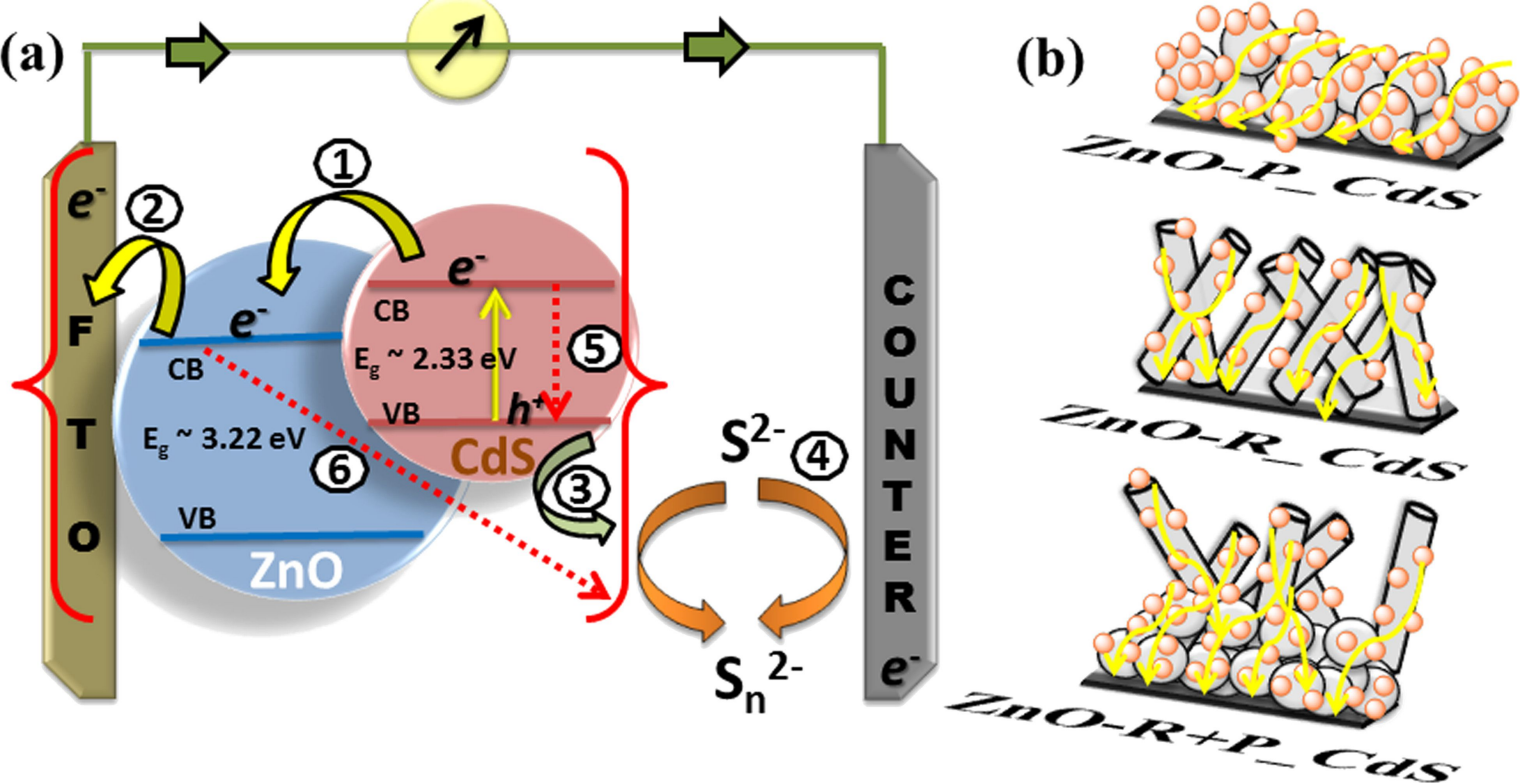
(a) and (b) Schematic representation of ZnO/CdS-based QDSSC cell basic operation principal and probable electron conduction pathways for colloidal CdS-sensitized ZnO-P, ZnO-P and ZnO-R+P-based solar cells.

An overall mechanism of electron transport in colloidal CdS ZnO based solar cells is illustrated schematically in Figure 9. After excitation, the basic electron conduction and regeneration throughout the cell involves the following steps (1) electron injection towards ZnO, (2) electron hopping towards FTO, (3) hole recombination, (4) electron regeneration (5) and (6) recombination of electron, as represented in Figure 9a. We have looked into the probable pathway for electron conduction through the different ZnO-based (P, R, R+P) photoanodes sensitized with colloidal CdS NPs, as schematically represented in Figure 9b. As stated previously, due to the higher surface area of the nanoparticles, more CdS was loaded on them than on the nanorods but uncontrolled agglomeration may result in random electron conduction and further may enhance the recombination rate. Whereas, the nanorods (due to their superior one-dimensional structure and reduced grain boundaries) are expected to favor a direct conduction pathway for rapid electron transport. In the case of ZnO-R+P based films, the higher CdS loading property of ZnO-P and the superior one-dimensional nature of ZnO-R has together enhanced the overall QDSSC performance compared to their individual systems.

In situ synthesis and deposition of QDs by the chemical bath deposition (CBD) or successive ionic layer adsorption and reaction (SILAR) method leads to the direct growth of QDs onto the electrode material surface by the chemical reaction of respective ionic species [48–50]. In order to understand the difference in the processing conditions, we further carried out experiments with the SILAR method also as shown in Table 1. In the CBD process, we have used the colloidal CdS synthesized by BSA templating which is anticipated to act as a functional linker to the electrode material surface. In spite of various optimizations in terms of varying the morphology of the used photoanodes, the use of surface functionalized CdS colloids, deposition techniques such as SILAR and CBD using the colloidal dispersion, and different types of electrolytes, the overall conversion efficiency of the fabricated cells could not be enhanced above 1%. However, the efficiency and the fill factor exhibited by our system is still comparable to most of the reports on ZnO/CdS systems. The probable reasons for the low efficiency and low FF in our systems are (i) presence of large number of unpassivated or partially passivated surface states as confirmed from emission characteristics and (ii) an ex situ colloidal CdS sensitization linking approach was implemented which has the advantage of controlling the properties of the CdS sensitizer through its size and shape, but may lead to more special distance between the QD and the photoelectrode surface and (iii) poor fill factors of the as-prepared DSSC devices.

## Conclusion

Colloidal CdS NPs have been synthesized through a bioinspired route using bovine serum albumin as a template. The structural, microstructural and optical studies confirmed the formation of phase pure cubic CdS NPs with average nanoparticle size of ≈5 nm with improved optical properties. The synthesized CdS NPs have been utilized as an alternative and cost-effective sensitizer for ZnO-based photoanodes. Various structural and microstructural analyses of the fabricated films further ascertain the successful sensitization of the synthesized colloidal CdS NPs. The photovoltaic performance has been monitored for the QDSSCs using ZnO nanoparticles, ZnO nanorods and a ZnO nanoparticle/nanorod mixture as photoanode materials and the synthesized CdS NPs as sensitizer. A maximum efficiency of 1.06% has been achieved in case of the ZnO-P+R based cell where ZnO-P acts as a blocking layer for better CdS sensitizing and ZnO-R has been introduced as a scattering layer for pronounced light harvesting.

## Supporting Information

File 1 Additional Experimental Information. Quantitative EDAX spectrum and zeta potential measurement of synthesized colloidal CdS, *I–V* characteristics CdS-sensitized ZnO-P, ZnO-R and ZnO-R+P films, emission data on films and a table with performance details on different electrolytes.

## References

[1] Rühle S, Shalom M, Zaban A (2010). ChemPhysChem.

[2] Tian J, Cao G (2013). Nano Rev.

[3] Salant A, Shalom M, Hod I, Faust A, Zaban A, Banin U (2010). ACS Nano.

[4] Kamat P V (2013). J Phys Chem Lett.

[5] Jun H K, Careem M A, Arof A K (2013). Renewable Sustainable Energy Rev.

[6] Kamat P V (2008). J Phys Chem C.

[7] Kim M R, Mar D (2015). J Phys Chem Lett.

[8] Tian J, Cao G (2013). Nano Rev.

[9] Duan J, Zhang H, Tang Q, He B, Yu L (2015). J Mater Chem A.

[10] Li C, Yang L, Xiao J, Wu Y-C, Søndergaard M, Luo Y, Li D, Meng Q, Iversen B B (2013). Phys Chem Chem Phys.

[11] Liu H, Zhang G, Sun W, Shen Z, Shi M (2015). PLoS One.

[12] Poornima K, Gopala Krishnan K, Lalitha B, Raja M (2015). Superlattices Microstruct.

[13] Ganesh T, Mane R S, Cai G, Chang J-H, Han S-H (2009). J Phys Chem C.

[14] Zhang Y, Xie T, Jiang J, Wei X, Pang S, Wang S, Wang D (2009). Nanotechnology.

[15] Qi J, Liu W, Biswas C, Zhang G, Sun L, Wang Z, Hu X, Zhang Y (2015). Opt Commun.

[16] Zhao Y, Guo H, Hua H, Xi Y, Hu C (2014). Electrochim Acta.

[17] Zhang P, Gao L (2003). Langmuir.

[18] Wei H H-Y, Evans C M, Swartz B D, Neukirch A J, Young J, Prezhdo O V, Krauss T D (2012). Nano Lett.

[19] Duxin N, Liu F, Vali H, Eisenberg A (2005). J Am Chem Soc.

[20] Ma N, Sargent E H, Kelley S O (2008). J Mater Chem.

[21] Baláž M, Baláž P, Sayagués M J, Zorkovská A (2013). Mater Sci Semicond Process.

[22] Tripathi R M, Bhadwal A S, Singh P, Shrivastav A, Singh M P, Shrivastav B R (2014). Adv Nat Sci: Nanosci Nanotechnol.

[23] Su H, Han J, Dong Q, Zhang D, Guo Q (2008). Nanotechnology.

[24] Naveenraj S, Asiri A M, Anandan S (2013). J Nanopart Res.

[25] Liang J-g, Ai X-p, He Z-k, Xie H-y, Pang D-w (2005). Mater Lett.

[26] Jhonsi M A, Kathiravan A, Renganathan R (2009). Colloids Surf, B.

[27] Na W, Liu X, Hu T, Su X (2016). New J Chem.

[28] Hagfeldt A, Boschloo G, Sun L, Kloo L, Pattersson H (2010). Chem Rev.

[29] Zhang Q, Dandeneau C S, Zhou X, Guozhong C (2009). Adv Mater.

[30] Lee W, Min S K, Dhas V, Ogale S B, Han S-H (2009). Electrochem Commun.

[31] Das P P, Agarkar S A, Mukhopadhyay S, Manju U, Ogale S B, Devi P S (2014). Inorg Chem.

[32] Das P P, Mukhopadhyay S, Agarkar S A, Jana A, Devi P S (2015). Solid State Sci.

[33] Das P P, Roy A, Das S, Devi P S (2016). Phys Chem Chem Phys.

[34] Lee Y-L, Chang C-H (2008). J Power Sources.

[35] Yang Z, Chen C-Y, Liu C-W, Chang H-T (2010). Chem Commun.

[36] Jiang J, He Y, Wan L, Cui Z, Cuia Z, Jessop P G (2013). Chem Commun.

[37] Dai Z, Zhang J, Bao J, Huang X, Mo X (2007). J Mater Chem.

[38] Malashchonak M V, Poznyak S K, Streltsov E A, Kulak A I, Korolik O V, Mazanik A V (2013). Beilstein J Nanotechnol.

[39] Mandal A, Saha J, De G (2011). Opt Mater.

[40] Murawala P, Phandis S M, Bhonde R R, Prasad B L V (2009). Colloids Surf, B.

[41] Jana A, Das P P, Agarkar S A, Devi P S (2014). Sol Energy.

[42] Uthirakumar P, Kang J H, Senthilarasu S, Hong C-H (2011). Physica E.

[43] Tak Y, Hong S J, Lee J S, Kijung Y (2009). J Mater Chem.

[44] Chen H, Li W, Liu H, Zhu L (2011). Electrochem Commun.

[45] Yao C-Z, Wei B-H, Meng L-X, Li H, Gong Q-J, Sun H, Ma H-X, Hu X-H (2012). J Power Sources.

[46] Luan C, Vaneski A, Susha A S, Xu X, Wang H-E, Chen X, Xu J, Zhang W, Lee C-S, Rogach A L (2011). Nanoscale Res Lett.

[47] Zhu G, Lv T, Pan L, Sun Z, Sun C (2011). J Alloys Compd.

[48] Hod I, González-Pedro V, Tachan Z, Fabregat-Santiago F, Mora-Seró I, Bisquert J, Zaban A (2011). J Phys Chem Lett.

[49] Seou M, Kim H, Tak Y, Yong K (2010). Chem Commun.

[50] Hodes G (2008). J Phys Chem C.

