# Supplementary material for: Performance of colloidal CdS sensitized solar cells with ZnO nanorods/nanoparticles
Source: Beilstein J Nanotechnol. 2017 Jan 23;8:210–21. doi: 10.3762/bjnano.8.23 (PMC5301656; doi:10.3762/bjnano.8.23)
Supplement: File 1 — Additional Experimental Information. Quantitative EDAX spectrum and zeta potential measurement of synthesized colloidal CdS, I–V characteristics CdS-sensitized ZnO-P, ZnO-R and ZnO-R+P films, emission data on films and a table with performance details on different electrolytes. [file Beilstein_J_Nanotechnol-08-210-s001.pdf]

Supporting Information

for

Performance of colloidal CdS sensitized solar cells with ZnO nanorods/nanoparticles

Anurag Roy[1,2], Partha Pratim Das[1,2], Mukta Tathavadekar[2,3], Sumita Das[1], and Parukuttyamma Sujatha Devi[1,2,*,§]

Address: [1]Sensor and Actuator Division, CSIR - Central Glass and Ceramic Research Institute, Kolkata 700032, India, [2]CSIR - Network Institute of Solar Energy (CSIR-NISE), New Delhi, India, and [3]Physical and Materials Chemistry Division, CSIR-National Chemical Laboratory, Pune 411 008, India

Email: Parukuttyamma Sujatha Devi - psujathadevi@cgcri.res.in

* Corresponding Author

§Tel.: +91-33-2483-8082; Fax: +91-33-2473-0957

Additional Experimental Information

Synthesis of ZnO rod and particle

Both the ZnO rod and particle has been prepared through aqueous solution grown process without addition of further any surfactant, templating agent or surface directing agent. In short ZnO rod has been synthesized through solution grown process according to our previously reported and 300°C calcined rod has been used for further studies[1]. Besides, ZnO particle has also been synthesized sonochemical way, in short 0.0375M Zn $(NO_3)_2$ as the precursor solution in water aqueous solution of 1 M NaOH was used as the precipitating agent. The aqueous solution of $Zn(NO_3)_2$ was sonicated for 2 hours using TAKASHI U1800-30kHz ultrasonic homogenizer followed by the addition of NaOH drop wise till the pH was reached 9±0.5. Then the precipitate was centrifuged at 10,000 rpm and washed thoroughly with water for two times to remove the impurities. After that it was dried in an oven at 60°C for 12 hours and finally the obtained products were calcined at 300°C.The resultant white powder was used for further studies.

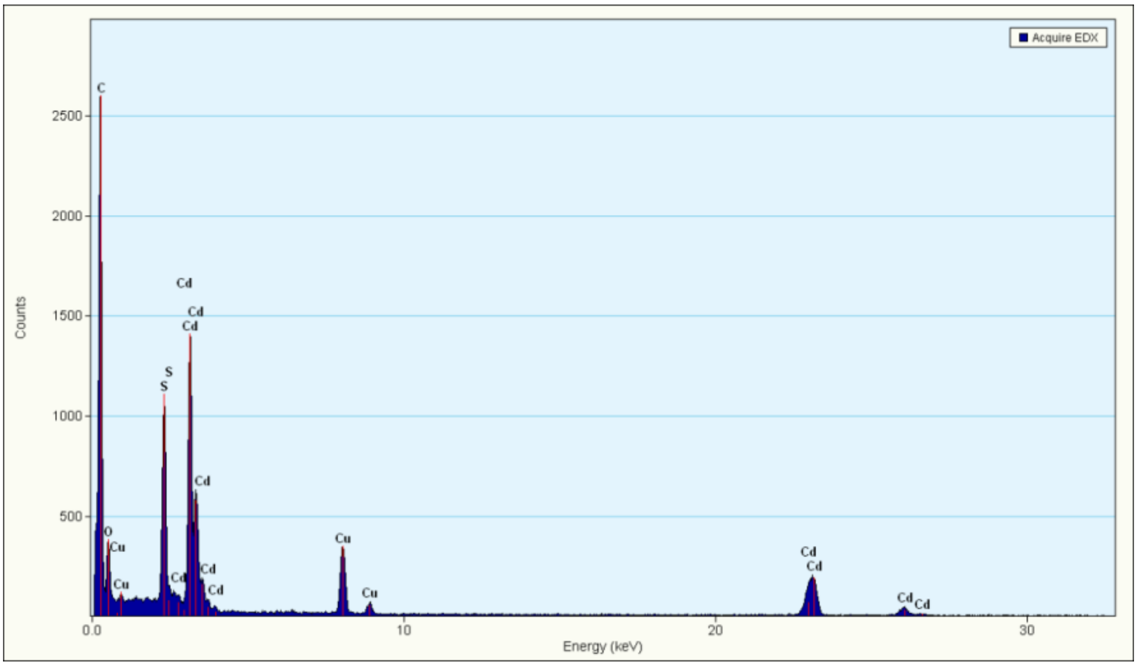

Element	Atomic(%)
S (K)	51.57
Cd (K)	48.42

Figure S1: Quantitative EDAX spectrum of the synthesized colloidal CdS.

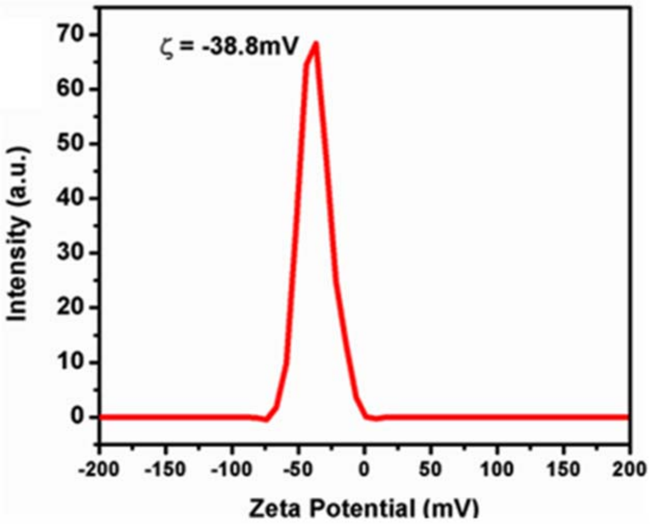

Figure S2: Zeta potential curve of the synthesized colloidal CdS.

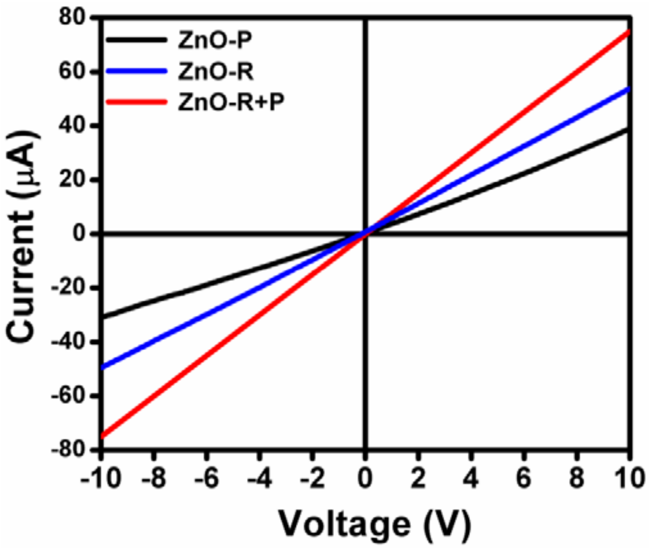

Figure S3: *I-V* characteristic plots for colloidal CdS sensitized ZnO-P, ZnO-R and ZnO-R+P based films.

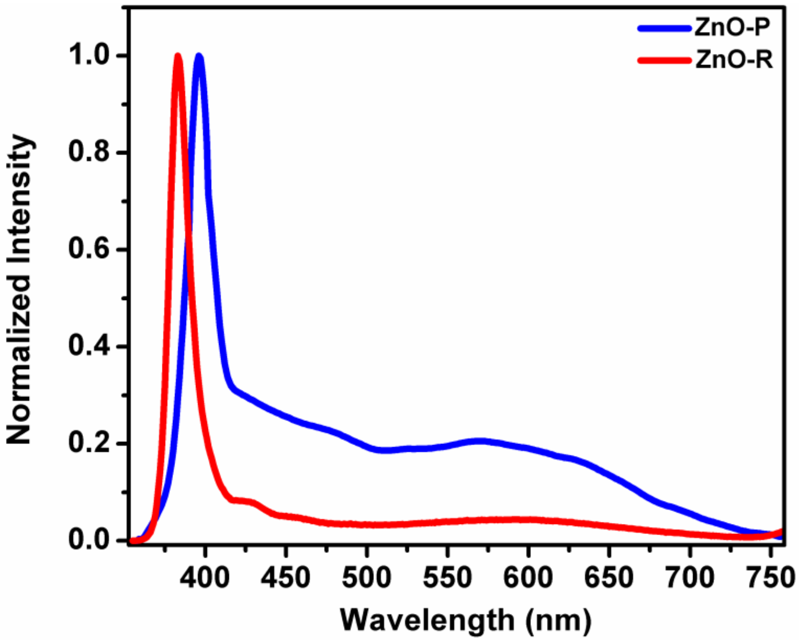

Figure S4: Room temperature photoluminescence spectra of ZnO-P and ZnO-R, excited at 345nm.

Table S1: Photovoltaic parameters of colloidal CdS sensitized ZnO cells using different electrolytes

**Cells**	**V_{OC} (V)**	**J_{SC} ($mA.cm^{-2}$)**	**FF (%)**	**Efficiency ± 0.02 (%)**	**Electrolyte used for colloidal CdS**	**Counter Electrode**	**Remarks**
ZnO-P	0.68	2.00	24.88	0.34	Polysulphide	Cu_xS	Colloidal BSA-CdS Ex-situ sensitization Process electrolyte and counter
ZnO-P	0.29	0.26	36.80	0.02	I^-/I^{3-}	Pt	
ZnO-R	0.66	3.48	34.13	0.80	Polysulphide	Cu_xS	
ZnO-R	0.35	0.28	40.65	0.04	I^-/I^{3-}	Pt	

							electrode varied

Reference

1. Das, P.P.; Agarkar, S.A.; Mukhopadhyay, S.; Manju, U.; Ogale, S.B.; Devi, P.S. *Inorg. Chem.***2014**, *53*, 3961-3972. doi: 10.1021/ic500279q
